# Detection and genetic diversity of *Bartonella* species in small mammals from the central region of the Qinghai-Tibetan Plateau, China

**DOI:** 10.1038/s41598-022-11419-x

**Published:** 2022-04-29

**Authors:** Juan Yu, Qingduo Li, Liang Lu, Shoujiang Li, Xiuping Song, Dongmei Li, Huaxiang Rao

**Affiliations:** 1 Department of Basic Medical Sciences, Changzhi Medical College, Changzhi, 046000 China; 2 Department of Public Health and Preventive Medicine, Changzhi Medical College, Changzhi, 046000 China; 3 State Key Laboratory for Infectious Disease Prevention and Control, Collaborative Innovation Center for Diagnosis and Treatment of Infectious Diseases, National Institute for Communicable Disease Control and Prevention, Chinese Center for Disease Control and Prevention, Beijing, 102206 China; 4 Institute for Communicable Disease Control and Prevention, Qinghai Center for Disease Control and Prevention, Xining, 810007 China

**Keywords:** Infectious-disease epidemiology, Bacterial genetics

## Abstract

In this study, we aimed to investigate the prevalence and molecular characteristics of *Bartonella* infections in small mammals from the central region of the Qinghai-Tibetan Plateau. Toward this, small mammals were captured using snap traps in Yushu City and Nangqian County, West China, and the spleen tissue was used for *Bartonella* culture. The suspected positive colonies were evaluated using polymerase chain reaction (PCR) amplification and by sequencing the citrate synthase (*gltA*) gene. We discovered that 31 out of the 103 small mammals tested positive for *Bartonella*, with an infection rate of 30.10%. Sex differences between the mammals did not result in a significant difference in infection rate (*χ*^2^ = 0.018, *P* = 0.892). However, there was a significant difference in infection rates in different small mammals (Fisher’s exact probability method, *P* = 0.017) and habitats (*χ*^2^ = 7.157, *P* = 0.028). Additionally, 31 *Bartonella* strains belonging to three species were identified, including *B*. *grahamii* (25), *B*. *japonica* (4) and *B*. *heixiaziensis* (2), among which *B*. *grahamii* was the dominant epidemic strain (accounting for 80.65%). Phylogenetic analyses showed that most of the *B*. *grahamii* isolates identified in this study may be closely related to the strains isolated from Japan and China. Genetic diversity analyses revealed that *B*. *grahamii* strains had high genetic diversity, which showed a certain host and geographical specificity. The results of Tajima’s test suggested that the *B*. *grahamii* followed the progressions simulated by a neutral evolutionary model in the process of evolution. Overall, a high prevalence and genetic diversity of *Bartonella* infection were observed in small mammals in the central region of the Qinghai-Tibetan Plateau. *B*. *grahamii* as the dominant epidemic strain may cause diseases in humans, and the corresponding prevention and control measures should be taken into consideration in this area.

## Introduction

*Bartonella* species are small, intracellular, vector-borne hemotrophic gram-negative bacteria. Thus far, there are over 40 species and subspecies have been reported to infect a wide range of mammals, including cats, dogs, rodents, bats, and so on^1^. Over 10 *Bartonella* species, including *B*. *bacilliformis*^2^, *B*. *quintana*^3^, *B*. *henselae*^4^, *B*. *elizabethae*^5^, *B*. *clarridgeiae*^6^, *B*. *koehlerae*^7^, *B*. *vinsonii* subsp. *arupensis*^8^, *B*. *vinsonii* subsp. *berkhoffii*^9^, *B*. *grahamii*^10,11^, *B*. *rochalimae*^12^, *B*. *tamiae*^13^, *B*. *ancashensis*^14^, *B*. *washoensis*^15^, can cause human diseases with various clinical manifestations, including periods of intermittent fever, and poly tissue inflammation involving the heart, liver, lymph nodes, and other tissues^16^. Small mammals, particularly rodents, are considered important reservoirs of *Bartonella* species, with an infection rate of 70% worldwide^17^. Hence, investigating the epidemiological characteristics of *Bartonella* in small mammals has important implications for the prevention and control of human bartonellosis.

The Qinghai-Tibetan Plateau, referred to as the "Roof of the World", is an inland plateau in Asia; the largest in China and the highest in the world. The Yushu Tibetan Autonomous Prefecture lies in the central region of the Qinghai-Tibetan Plateau and belongs to the Sanjiangyuan Region, the source of the Yangtze, Yellow, and Lantsang rivers (between 31.65° and 36.27° N, 89.40°  and  102.38° E), with an average elevation of 4493 m^18^. It has an important ecological status, with the highest concentration of biodiversity area in the world; nearly 30 species of mammals have been reported to inhabit this area. Our team has previously detected *Bartonella* species infection in small mammals in some areas of the Qinghai-Tibetan Plateau, with infection rates of 18.99% and 38.61%^19,20^. However, investigations of *Bartonella* species in small mammals in the central region of the Qinghai-Tibetan Plateau have not yet been undertaken. This region’s tourism industry was greatly developed following the reconstruction work after the Yushu earthquake. This increased the probability of people being infected with natural infectious diseases. Therefore, in this study, we investigated the prevalence and genetic diversity of *Bartonella* species in small mammals in the Yushu Tibetan Autonomous Prefecture. Our findings provide insights into the distribution and genetic diversity of *Bartonella* in small mammals and the scientific basis for the control and prevention of *Bartonella* infection in humans in this region.

## Results

### Animal collection

**Figure 1:**
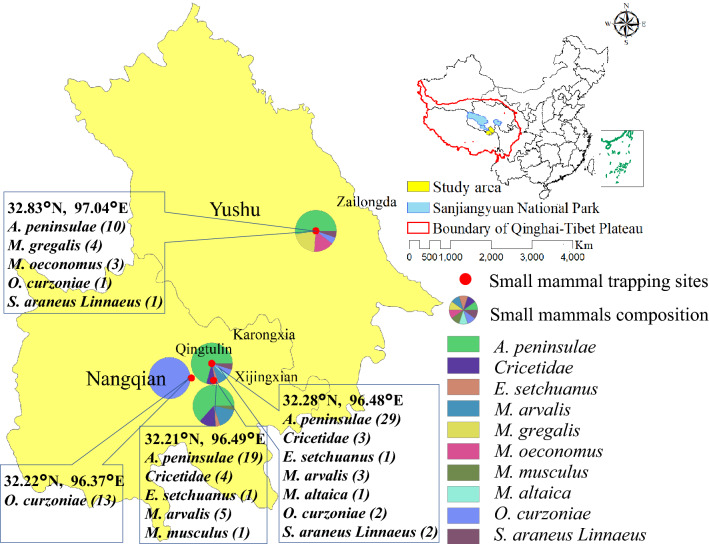
Geographical distribution of the trapped small mammals in the central region of the Qinghai-Tibetan Plateau, China. The map was prepared in ArcGIS 10.2.2 using political boundaries from the National Geomatics Center of China (http://www.ngcc.cn/ngcc) for illustrative purposes only, these data are available free of charge.

A total of 103 small mammals were captured and categorized into 10 species based on their morphology, including *Apodemus peninsulae* (58), *Ochotona curzoniae* (16), *Microtus arvalis* (8), *Cricetidae* (7), *Microtus gregalis* (4), *Microtus oeconomus* (3), *Sorex araneus Linnaeus* (3), *Eozapus setchuanus* (2), *Mustela altaica* (1), and *Mus musculus* (1). The geographical distribution of the trapped small mammals is shown in Fig. 1.

### Bartonella infections

Spleens of the small mammals were collected and used for *Bartonella* isolation, and the pure colonies obtained were confirmed by polymerase chain reaction (PCR) amplification of the partial citrate synthase (*gltA*) gene (379 bp). In total, 31 small mammals were positive for *Bartonella* infection, with an infection rate of 30.10% (31/103), which were classified into five species (*Apodemus peninsulae* (22/58), *Microtus arvalis* (2/8), *Cricetidae* (4/7), *Microtus gregalis* (1/4), *Microtus oeconomus* (2/3)). The difference of infection rate among different small mammals was significant (Fisher’s exact probability method, *P* = 0.017) (Table 1).

**Table 1:** Distribution of *Bartonella* infection in different small mammals.

Host	*n*	No. PCR positive (%)	*B*. *grahamii* (%)	*B*. *japonica* (%)	B. *heixiaziensis* (%)
AP	58	22 (37.93)	18 (31.03)	4 (6.90)	0 (0.00)
OC	16	0 (0.00)	0 (0.00)	0 (0.00)	0 (0.00)
MA	8	2 (25.00)	1 (12.50)	0 (0.00)	1 (12.50)
CR	7	4 (57.14)	4 (57.14)	0 (0.00)	0 (0.00)
MG	4	1 (25.00)	1 (25.00)	0 (0.00)	0 (0.00)
MO	3	2 (66.67)	1 (33.33)	0 (0.00)	1 (33.33)
SA	3	0 (0.00)	0 (0.00)	0 (0.00)	0 (0.00)
ES	2	0 (0.00)	0 (0.00)	0 (0.00)	0 (0.00)
MuA	1	0 (0.00)	0 (0.00)	0 (0.00)	0 (0.00)
MuM	1	0 (0.00)	0 (0.00)	0 (0.00)	0 (0.00)
Total	103	31 (30.10)	25 (24.27)	4 (3.88)	2 (1.94)

AP *Apodemus peninsulae*, OC *Ochotona curzoniae*, MA *Microtus arvalis*, CR *Cricetidae*, MG *Microtus gregalis*, MO *Microtus oeconomus*, SA *Sorex araneus Linnaeus*, ES *Eozapus setchuanus*, MuA *Mustela altaica*, MuM *Mus musculus.*

Of the 103 small mammals, 42 were male, 50 were female, and 11 had no sex information. The infection rate was 32.00% (16/50) in females and 33.33% (14/42) in males, and the difference was not significant (*χ*^2^ = 0.018, *P* = 0.892). Forty-nine small mammals—corresponding to nine species—were captured in farmlands, with a *Bartonella* infection rate of 30.61% (15/49). Forty-one small mammals—corresponding to seven species—were captured in forests with an infection rate of 39.02% (16/41). Additionally, 13 small mammals of the same species were captured in meadows with no *Bartonella* infection. Thus, the infection rates between different habitats were significantly different (*χ*^2^ = 7.157, *P* = 0.028) (Tables 2, 3).

**Table 2:** Positive rate of *Bartonella* infection of small mammals in different habitats.

Habitats	Host										No. captured	No. PCR positive	Positive rate (%)
	AP	OC	MA	CR	MG	MO	SA	ES	MuA	MuM			
Farmland	29	1	5	4	4	3	1	1	0	1	49	15	30.61
Forest	29	2	3	3	0	0	2	1	1	0	41	16	39.02
Meadow	0	13	0	0	0	0	0	0	0	0	13	0	0.00
Total	58	16	8	7	4	3	3	2	1	1	103	31	30.10

**Table 3:** Sampling locations of each host species with *Bartonella* infection.

Sample ID	Host species	Sex	Habitat	Location	Latitude	Longitude	Genotype
AP1QHYS	*Apodemus peninsulae*	Male	Farmland	Zailongda	32.83° N	97.04° E	*Bartonella grahamii*
MO2QHYS	*Microtus oeconomus*	Female	Farmland	Zailongda	32.83° N	97.04° E	*Bartonella heixiaziensis*
MO4QHYS	*Microtus oeconomus*	Male	Farmland	Zailongda	32.83° N	97.04° E	*Bartonella grahamii*
MG5QHYS	*Microtus gregalis*	Unknown	Farmland	Zailongda	32.83° N	97.04° E	*Bartonella grahamii*
AP11QHYS	*Apodemus peninsulae*	Male	Farmland	Zailongda	32.83° N	97.04° E	*Bartonella japonica*
AP14QHYS	*Apodemus peninsulae*	Female	Farmland	Zailongda	32.83° N	97.04° E	*Bartonella grahamii*
AP16QHYS	*Apodemus peninsulae*	Male	Farmland	Zailongda	32.83° N	97.04° E	*Bartonella grahamii*
CR34QHYS	*Cricetidae*	Female	Farmland	Xijingxian	32.21° N	96.49° E	*Bartonella grahamii*
CR36QHYS	*Cricetidae*	Female	Farmland	Xijingxian	32.21° N	96.49° E	*Bartonella grahamii*
AP37QHYS	*Apodemus peninsulae*	Male	Farmland	Xijingxian	32.21° N	96.49° E	*Bartonella grahamii*
AP39QHYS	*Apodemus peninsulae*	Female	Farmland	Xijingxian	32.21° N	96.49° E	*Bartonella grahamii*
AP43QHYS	*Apodemus peninsulae*	Male	Farmland	Xijingxian	32.21° N	96.49° E	*Bartonella grahamii*
AP51QHYS	*Apodemus peninsulae*	Female	Farmland	Xijingxian	32.21° N	96.49° E	*Bartonella japonica*
MA60QHYS	*Microtus arvalis*	Female	Farmland	Xijingxian	32.21° N	96.49° E	*Bartonella heixiaziensis*
MA61QHYS	*Microtus arvalis*	Female	Farmland	Xijingxian	32.21° N	96.49° E	*Bartonella grahamii*
AP63QHYS	*Apodemus peninsulae*	Female	Forest	Karongxia	32.28° N	96.48° E	*Bartonella grahamii*
AP66QHYS	*Apodemus peninsulae*	Male	Forest	Karongxia	32.28° N	96.48° E	*Bartonella grahamii*
AP67QHYS	*Apodemus peninsulae*	Female	Forest	Karongxia	32.28° N	96.48° E	*Bartonella grahamii*
AP69QHYS	*Apodemus peninsulae*	Male	Forest	Karongxia	32.28° N	96.48° E	*Bartonella grahamii*
AP70QHYS	*Apodemus peninsulae*	Female	Forest	Karongxia	32.28° N	96.48° E	*Bartonella grahamii*
AP71QHYS	*Apodemus peninsulae*	Female	Forest	Karongxia	32.28° N	96.48° E	*Bartonella grahamii*
AP74QHYS	*Apodemus peninsulae*	Male	Forest	Karongxia	32.28° N	96.48° E	*Bartonella grahamii*
AP76QHYS	*Apodemus peninsulae*	Male	Forest	Karongxia	32.28° N	96.48° E	*Bartonella grahamii*
AP79QHYS	*Apodemus peninsulae*	Male	Forest	Karongxia	32.28° N	96.48° E	*Bartonella grahamii*
AP95QHYS	*Apodemus peninsulae*	Male	Forest	Karongxia	32.28° N	96.48° E	*Bartonella grahamii*
AP96QHYS	*Apodemus peninsulae*	Female	Forest	Karongxia	32.28° N	96.48° E	*Bartonella japonica*
AP98QHYS	*Apodemus peninsulae*	Female	Forest	Karongxia	32.28° N	96.48° E	*Bartonella japonica*
AP100QHYS	*Apodemus peninsulae*	Female	Forest	Karongxia	32.28° N	96.48° E	*Bartonella grahamii*
AP101QHYS	*Apodemus peninsulae*	Male	Forest	Karongxia	32.28° N	96.48° E	*Bartonella grahamii*
CR102QHYS	*Cricetidae*	Female	Forest	Karongxia	32.28° N	96.48° E	*Bartonella grahamii*
CR103QHYS	*Cricetidae*	Male	Forest	Karongxia	32.28° N	96.48° E	*Bartonella grahamii*

### Identification of *Bartonella* species

Through BLAST analysis of the *gltA* gene, 25 isolates were identified to be *B*. *grahamii* with 97.11–100.00% identity, including 18 isolates from *A*. *peninsulae*, 4 isolates from *Cricetidae*, 1 isolate from *M*. *arvalis*, 1 isolate from *M*. *gregalis* and 1 isolate from *M*. *oeconomus*; 4 isolates from *A*. *peninsulae* were *B*. *japonica* with 97.89–99.70% identity; 2 isolates were *B*. *heixiaziensis* with 98.59–99.44% identity, including 1 isolate from *M*. *arvalis* and 1 isolate from *M*. *oeconomus* (Table 1).

**Figure 2:**
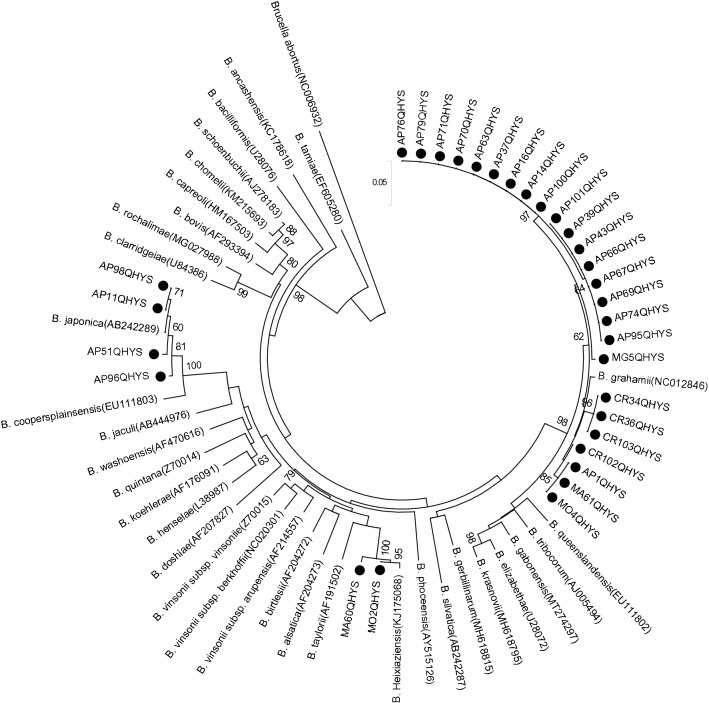
Phylogenetic trees constructed based on *gltA* gene of 31 *Bartonella* isolates. The tree was constructed by using the maximum-likelihood (ML) method with the Kimura 2-parameter model, bootstrap values calculated with 1000 replicates in MEGA version 7.0 (https://www.megasoftware.net). The sequences detected in this study are indicated with black dots. *Brucella abortus* was used as outgroup.

**Figure 3:**
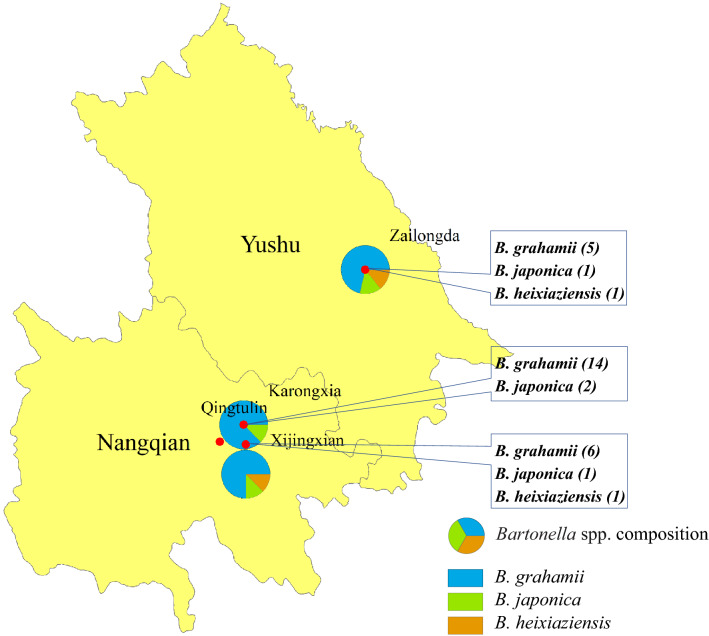
*Bartonella* species composition in different sampling sites in the central region of the Qinghai-Tibetan Plateau, China. The map was prepared in ArcGIS 10.2.2 using political boundaries from the National Geomatics Center of China (http://www.ngcc.cn/ngcc) for illustrative purposes only, these data are available free of charge.

In our previous study, phylogenetic analyses of *Bartonella* species was performed based on the DNA sequences of the *gltA*, *ftsZ*, *rpoB and ribC* revealed the same results^20^. Of these, *gltA* is the most commonly used in the phylogenetic analyses of *Bartonella*. Therefore, in this study, we selected *gltA* to construct a phylogenetic tree using the maximum likelihood (ML) method. All *Bartonella* strains could be divided into three clusters, i.e., *B*. *grahamii*, *B*. *heixiaziensis*, and *B*. *japonica*; *B*. *grahamii* were the dominant *Bartonella* species in this area (Fig. 2). *Bartonella* was detected in small mammals from three of the four trapping sites and the distribution of *Bartonella* species showed slight geographical differences (Fig. 3).

**Figure 4:**
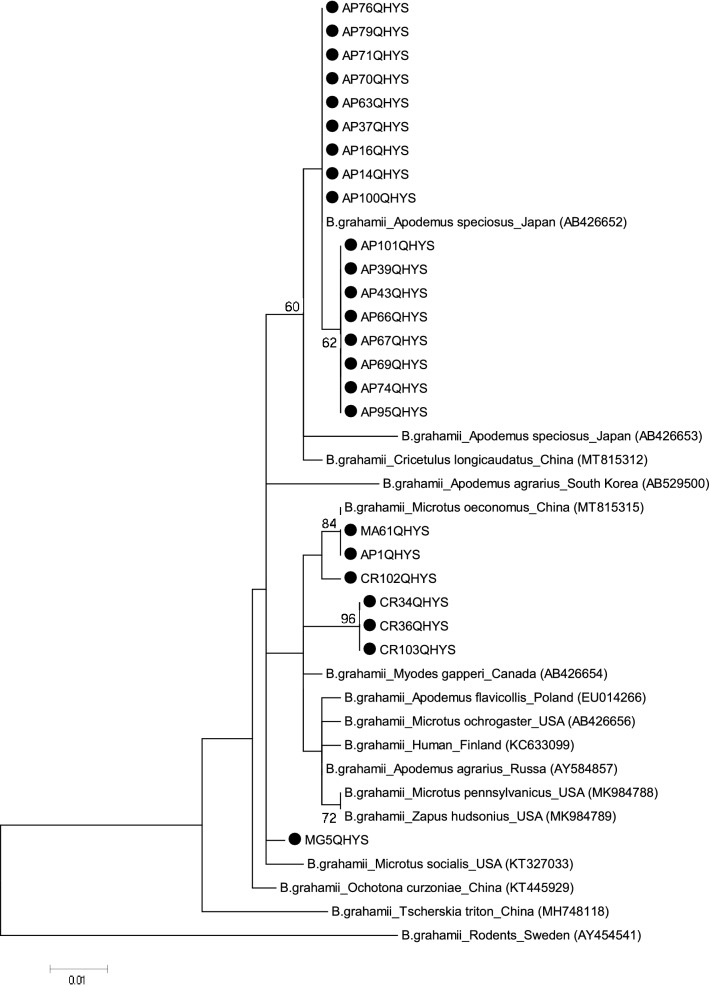
Traceability analyses of *B*. *grahamii* based on *gltA* gene. The tree was constructed by using the maximum-likelihood (ML) method with the Kimura 2-parameter model, bootstrap values calculated with 1000 replicates in MEGA version 7.0 (https://www.megasoftware.net). The sequences detected in this study are indicated with black dots.

Phylogenetic analyses based on *gltA* sequences showed that *B*. *grahamii* was mainly grouped into four clusters, indicating that *B*. *grahamii* might have the different origins. We then obtained the *gltA* sequences of *B*. *grahamii* from GenBank released before July, 2021, and performed the traceability analyses. The majority of *B*. *grahamii* strains from *A*. *peninsulae* clustered with *B*. *grahamii* from *A*. *speciosus* in Japan; three strains, i.e., AP1QHYS, MA61QHYS, CR102QHYS, from *A*. *peninsulae*, *M*. *arvalis*, and *Cricetidae* clustered with *B*. *grahamii* from *M*. *oeconomus* in our previous study; three strains, i.e., CR34QHYS, CR36QHYS, CR103QHYS, from *Cricetidae* and one strain from *M*. *gregalis* (MG5QHYS) clustered separately and not with the reference strains (Fig. 4).

### Genetic diversity analyses

**Figure 5:**
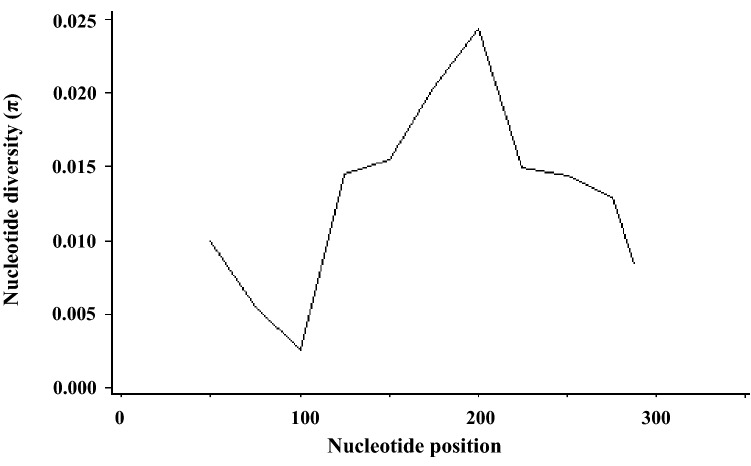
Genetic diversity of different nucleotide position in *gltA* gene of *B*. *grahamii*. Genetic diversity was analyzed using DNASP 6.12.03 (http://www.ub.edu/dnasp) with a sliding window interval of 25 bp.

Subsequently, the genetic diversity of the *gltA* gene sequence (326 bp) from 77 strains of *B*. *grahamii* was analyzed, including 25 strains in this study and 52 strains from our previous studies, isolated from three regions of Qinghai-Tibetan Plateau, including Haixi Mongolian and Tibetan Autonomous Prefecture, Huangnan Tibetan Autonomous Prefecture, and Haibei Tibetan Autonomous Prefecture. We found 19 polymorphic loci (S = 19) and 15 haplotypes (H = 15). The haplotype diversity (Hd) was 0.880 ± 0.019, the mean number of nucleotide differences (κ) was 4.386, and the nucleotide diversity (π) was 0.01345 ± 0.00077. DNA polymorphism was analyzed using a sliding window with a length of 100 bp and a step size of 25 bp. It was found that fragment diversity was the highest between 151 and 250 bp (Fig. 5). The results indicated high genetic diversity in *B*. *grahamii* in this area. Tajima's D was calculated as 0.39958 (*P* > 0.10), suggesting that *B*. *grahamii* followed the progressions simulated by a the neutral evolutionary model in the process of evolution.

**Figure 6:**
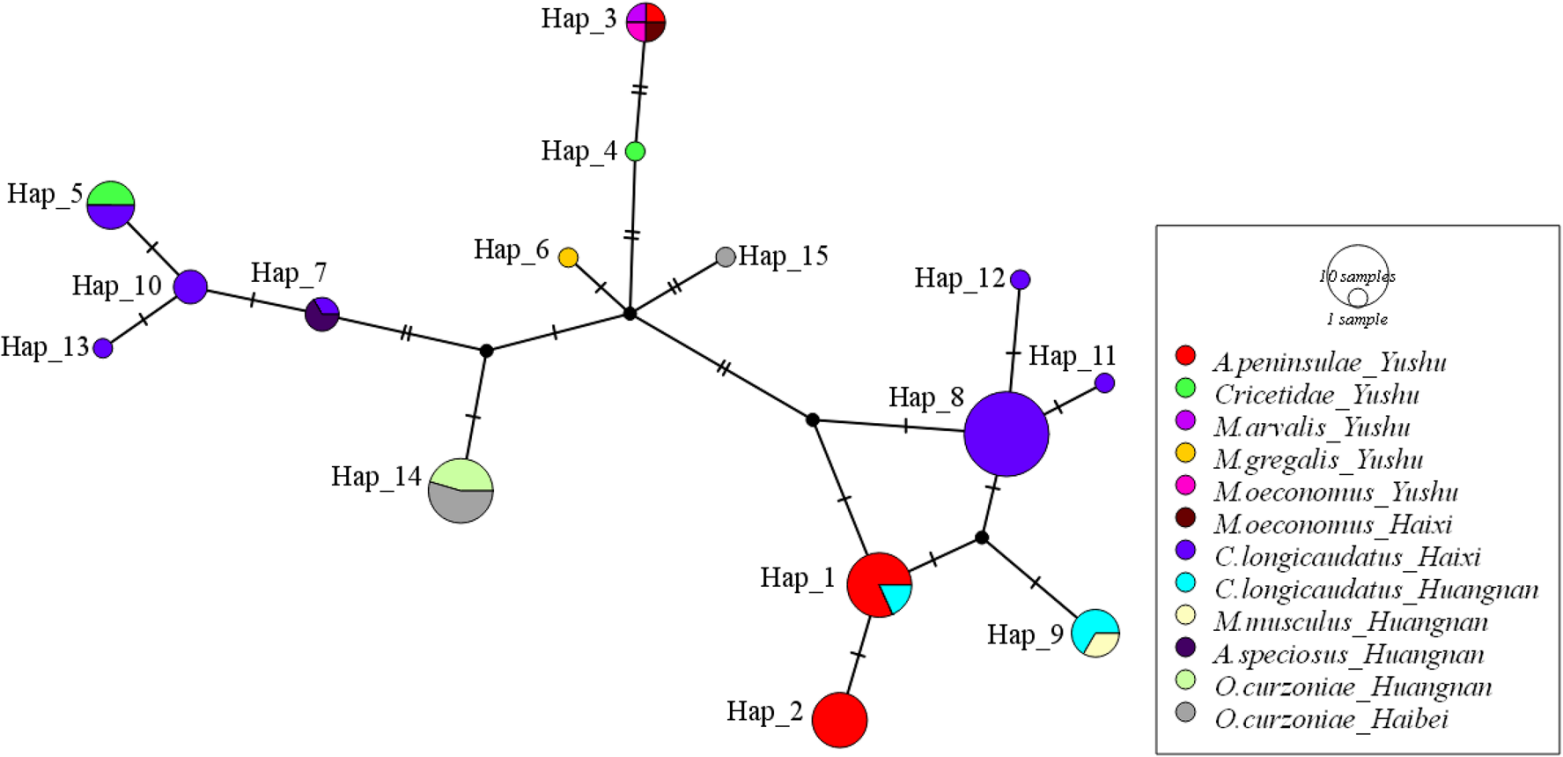
Median-joining networks of *gltA* gene for *B. grahamii* strains from different hosts and regions in the Qinghai-Tibetan Plateau, China. The sequences were analyzed based on a median-joining network using the Population Analysis with Reticulate Trees (PopART) software version 1.7 (http://popart.otago.ac.nz/index.shtml) with the default setting (epsilon = 0).

Haplotype network analyses showed that 35 strains from *Cricetulus longicaudatus* contained 10 haplotypes (2 strains for Hap 1, 3 strains for Hap 5, 1 strain for Hap 7, 19 strains for Hap 8, 4 strains for Hap 9, 3 strains for Hap 10, 1 strain for Hap 11, 1 strain for Hap 12 and 1 strain for Hap 13), 18 strains from *A. peninsulae* contained 3 haplotypes (9 strains for Hap 1, 8 strains for Hap 2, and 1 strain for Hap 3), 12 strains from *O. curzoniae* contained 2 haplotypes (11 strains for Hap 14 and 1 strain for Hap 15), 4 strains from *Cricetidae* contained 2 haplotypes (1 strain for Hap 4 and 3 strains for Hap 5), 2 strains from *Apodemus speciosus* for Hap 7, 2 strains from *M. musculus* for Hap 9, 1 strain from *M. arvalis* and 2 strains from *M. oeconomus* for Hap 3, and 1strain from *M. gregalis* for Hap 6. In addition, 25 strains isolated from Yushu contained 6 haplotypes (9 strains for Hap 1, 8 strains for Hap 2, 3 strains for Hap 3, 1 strain for Hap 4, 3 strains for Hap 5, and 1 strain for Hap 6), 30 strains isolated from Haixi contained 8 haplotypes (1 strain for Hap 3, 3 strains for Hap 5, 1 strain for Hap 7, 19 strains for Hap 8, 3 strains for Hap 10, 3 strains for Hap 11, 12, 13 respectively), 15 strains isolated from Huangnan contained 4 haplotypes (2 strains for Hap 1, 2 strains for Hap 7, 6 strains for Hap 9, and 5 strains for Hap 14), and 7 strains isolated from Haibei contained 2 haplotypes (6 strains for Hap 14 and 1 strain for Hap 15) (Fig. 6, Table 4).

**Table 4:** Haplotypes of *B. grahamii* strains.

Haplotype	Sample ID and NCBI accession number
Hap-1	AP100QHYS AP14QHYS AP16QHYS AP37QHYS AP63QHYS AP70QHYS AP71QHYS AP76QHYS AP79QHYS CL18QHHN(MT821838) CL3QHHN(MT821823)
Hap-2	AP101QHYS AP39QHYS AP43QHYS AP66QHYS AP67QHYS AP69QHYS AP74QHYS AP95QHYS
Hap-3	AP1QHYS MA61QHYS MO4QHYS MO20QHHX(MT815315)
Hap-4	CR102QHYS
Hap-5	CR103QHYS CR34QHYS CR36QHYS CL19QHHX(MT815290) CL65QHHX(MT815306) CL73QHHX(MT815311)
Hap-6	MG5QHYS
Hap-7	AS10QHHN(MT821832) AS19QHHN(MT821840) CL68QHHX(MT815308)
Hap-8	CL01QHHX(MT815286) CL03QHHX(MT815287) CL05QHHX(MT815288) CL09QHHX(MT815289) CL25QHHX(MT815291) CL26QHHX(MT815292) CL27QHHX(MT815293) CL29QHHX(MT815294) CL32QHHX(MT815295) CL33QHHX(MT815296) CL34QHHX(MT815297) CL41QHHX(MT815298) CL42QHHX(MT815299) CL43QHHX(MT815300) CL45QHHX(MT815301) CL46QHHX(MT815302) CL48QHHX(MT815303) CL50QHHX(MT815304) CL70QHHX(MT815310)
Hap-9	CL1QHHN(MT821820) CL2QHHN(MT821822) CL6QHHN(MT821826) CL7QHHN(MT821828) MM12QHHN(MT821834) MM15QHHN(MT821837)
Hap-10	CL64QHHX(MT815305) CL69QHHX(MT815309) CL75QHHX(MT815313)
Hap-11	CL67QHHX(MT815307)
Hap-12	CL74QHHX(MT815312)
Hap-13	CL76QHHX(MT815314)
Hap-14	OC01QHHB(KT445915) OC03QHHB(KT445917) OC07QHHB(KT445917) OC29HBQH(KT445920) OC41HBQH(KT445923) OC42QHHB(KT445924) OC66QHHN(KT445925)OC68QHHN(KT445926) OC71QHHN(KT445927) OC73QHHN(KT445928) OC74QHHN(KT445929)
Hap-15	OC19QHHB(KT445918)

AP *Apodemus peninsulae*, AS *Apodemus speciosus,* CL *Cricetulus longicaudatus,* CR *Cricetidae*, MA *Microtus arvalis*, MG *Microtus gregalis*, MO *Microtus oeconomus*, MM *Mus musculus,* OC *Ochotona curzoniae*. QH Qinghai Province, HB Haibei, HN Huangnan, HX Haixi, YS Yushu.

## Discussion

*Bartonella* species are distributed throughout the world. They are highly prevalent in small mammals and are generally transmitted by bloodsucking arthropod vectors^21^. Previous studies have revealed that *Bartonella* infection varies in different regions and animals^16,17^. For instance, infection rate of *Bartonella* in rodents is 4–50% in China^22,23^, 6–94% in Japan^24,25^, 7–14% in Korea^26^, 2–10% in Indonesia^27^, 60–83% in Russia^28^, 6–90% in United States^29,30^, 20–60% in England^31,32^. Rodents are primary reservoir hosts for *B*. *grahamii*^33^, *B*. *elizabethae*^34^, and *B*. *vinsonii* subsp. *arupensis*^35^; domestic cats are primary reservoir hosts for *B*. *henselae*^36^, *B*. *clarridgeiae*^37^, and *B*. *koehlerae*^38^; dogs are primary reservoir hosts for *B*. *henselae* and *B*. *vinsonii* subsp. *berkhoffii*^39^. Therefore, it is necessary to investigate the *Bartonella* infections in small mammals from different areas.

In this study, we observed the prevalence and molecular characteristics of *Bartonella* species in small mammals from the central region of the Qinghai-Tibetan Plateau. The infection rate of *Bartonella* species in small mammals was 30.10%, which was similar to that of 38.61% in Qaidam Basin as determined in our previous study^20^, and higher than that in most areas of China^40^. *Bartonella* species were detected in five out of ten species of small mammals, including *A*. *peninsulae*, *M*. *arvalis*, *Cricetidae*, *M*. *gregalis*, and *M*. *oeconomus*, and we found differing infection rates among them. Additionally, the infection rate varied significantly by habitats, but not by sex, which is concurrent with the results of a previous study^20^.

*Bartonella* species are fastidious, slow-growing, facultative intracellular bacteria that are difficult and time consuming to culture. In our previous study, we used spleen, liver, and brain tissue for *Bartonella* culture and found that the positive rates in different tissues of small mammals did not differ significantly^19,20^. Here, the spleen tissue of small mammals was used for *Bartonella* culture, and 31 *Bartonella* strains were obtained. BLAST and phylogenetic analyses showed that 31 *Bartonella* strains corresponded to three species of *Bartonella—*(*B*. *grahamii*, *B*. *japonica*, and *B*. *heixiaziensis*). Importantly, 80.65% isolates (25/31) were *B*. *grahamii* and detected in all five species of the small mammals studied, suggesting that it was the dominant *Bartonella* strain. *B*. *grahamii* is associated with neuroretinitis and cat scratch disease (CSD) in immunocompromised individuals^10,11^, suggesting that *Bartonella* species may have the ability to cause human disease in this area. In addition, four isolates of *B*. *japonica* were isolated from *A*. *peninsulae* and two isolates of *B*. *heixiaziensis* were isolated from *Microtus* species, indicating specificity of infection among rodent species.

Subsequently, we performed traceability analyses on the dominant *B*. *grahamii* strains and found that *B*. *grahamii* was mainly grouped into four clusters. *B. grahamii* was clustered with *A*. *speciosus* in Japan, *M*. *oeconomus* in China, 3 strains from *Cricetidae* and 1 strain from *M*. *gregalis* clustered separately. These results indicated that *B*. *grahamii* might have different origins. Further studies are needed to determine whether the pathogenicity of *B*. *grahamii* strains differs depending on their origins.

A previous study revealed that the polymorphism within *gltA* gene was high in *Bartonella* species^33^. Here, 15 haplotypes were detected in 77 strains of *B. grahamii* based on *gltA* gene (Hd = 0.880, *π* = 0.01345), suggesting that the high genetic diversity of *B. grahamii* in the Qinghai-Tibetan Plateau. With the exception of *C. longicaudatus*, *B. grahamii* strains were isolated from *A. peninsulae* for Hap 1–3, *Cricetidae* for Hap 4–5, *M. arvalis* and *M. oeconomus* for Hap 3, *M. musculus* for Hap 9, and *O. curzoniae* for Hap 14–15, which suggested the haplotypes of *B. grahamii* showed a certain host specificity. This also indicated the haplotypes of *B. grahamii* had a certain geographical specificity, although, the geographical crossover existed. Additionally, *B. grahamii* isolated from *C. longicaudatus* showed complex haplotypes that intersected with many rodents, suggesting that it might be important for the evolution of *B. grahamii* in this area.

## Conclusions

In conclusion, *Bartonella* infection rate was 30.10% in small mammals in the central region of the Qinghai-Tibetan Plateau, with significant differences between different animal species and habitats. *B*. *grahamii*, *B*. *japonica*, and *B*. *heixiaziensis* were detected in five rodent species, *A*. *peninsulae*, *M*. *arvalis*, *Cricetidae*, *M*. *gregalis* and *M*. *oeconomus*. *B*. *grahamii* was the dominant strain, and originated from the *B*. *grahamii* strains in different areas. In addition, high genetic diversity in *B*. *grahamii* was observed in this area, and the haplotypes of *B. grahamii* showed a certain host and geographical specificity. Our results further enrich the prevalence and molecular characteristics of *Bartonella* infection in small mammals in the Qinghai-Tibetan Plateau, which could provide the scientific basis for prevention and control of rodent-*Bartonella* species.

## Materials and methods

### Animal collection

Small mammals were captured using snap traps in July 2019 in Yushu City (32.68°–33.77° N, 95.68°–97.73° E) and Nangqian County (31.53°–32.72° N, 95.35°–97.12° E) of Qinghai Province, which were identified morphologically.

### Bartonella culture

Spleens were harvested under sterile conditions from each animal after euthanasia. Approximately 20 mg of each spleen sample was homogenized by adding 200 μL sterilized trypsin soy broth (BD Biosciences, Franklin Lakes, NJ, USA), plated onto two trypsin soy agars containing 5% (vol/vol) defiber sheep blood (BD Biosciences), and incubated at 37 °C in an atmosphere containing 5% CO_2_. Pure *Bartonella* colonies were obtained using a protocol described in previous studies^19^.

### DNA extraction, PCR amplification and DNA sequencing

Crude DNA was extracted using a previously reported method^19^. PCR was performed to detect the *Bartonella gltA* gene. DNA amplification was performed in 50 μL mixtures containing 25 μL 2 × TransTaq-T PCR SuperMix (Beijing TransGen Biotech Co., Ltd., Beijing, China), 22 μL double-distilled H_2_O, 1 μL (10 μmol/L) of each primer (BhCS781.p: GGGGACCAGCTCATGGTGG; BhCS1137.n: AATGCAAAAAGAACAGTAAACA^41^), and 1 μL of DNA template. *gltA* amplification was performed under the following conditions: one cycle for 5 min at 94 °C; 33 cycles for 30 s at 94 °C, 30 s at 53 °C, and 20 s at 72 °C; and a final extension for 7 min at 72 °C. Next, 5 μL of each PCR product was run on 1% agarose gels, stained with ethidium bromide, and visualized using a gel imaging system (Bio-Rad, Hercules, CA, USA). The expected PCR products were purified using the QIAquick PCR Purification Kit (Qiagen, Hilden, Germany) according to the manufacturer’s protocols, and then sequenced on an Applied Biosystems 3730 xl Genetic Analyzer (Applied Biosystems, Foster City, CA, USA).

### Phylogenetic analyses

The sequences generated in this study have been submitted to GenBank (accession numbers MZ126613-MZ126643). The nucleotide sequences of the isolated sequences were compared against the *Bartonella* species sequences hosted on GenBank using BLAST at the National Center for Biotechnology Information Website (http://blast.ncbi.nlm.nih.gov/Blast.cgi). The *gltA* sequences of *B*. *grahamii* hosted on GenBank released before July, 2021 were collected for traceability analyses. Furthermore, one strain isolated from the same host in the same laboratory at the same time was selected randomly as the reference strain. A phylogenetic tree was created using the maximum-likelihood method with the Kimura 2-parameter model in MEGA version 7.0, and bootstrap values were calculated with 1000 replicates^42,43^. *Brucella abortus* was used as the outgroup.

### Genetic diversity analyses

The polymorphism of nucleotide sequences, including the number of polymorphic sites (S), the number of haplotypes (H), the nucleotide diversity (π), the mean number of nucleotide differences (κ), and the haplotype diversity (Hd), was analyzed using DNASP 6.12.03. A sliding window interval of 25 bp was used to determine which segment of the target gene sequence had the highest nucleotide diversity (π) by analyzing 100 bp each time. Tajima’s test was performed to determine whether the target gene sequence followed the progressions simulated by a neutral evolutionary model in the process of evolution. Then, the sequences were analyzed based on a median-joining network using the Population Analysis with Reticulate Trees (PopART) software version 1.7 with the default setting (epsilon = 0).

### Statistical analysis

The positive rates of *Bartonella* in different habitats and sexes of small mammals were analyzed using the Chi-square test. The infection rates of *Bartonella* in different mammals were analyzed using the Fisher’s exact probability method. All data were analyzed using SPSS 22.0 (SPSS, Inc., Chicago, IL, USA). Significance was set at *P* < 0.05.

### Ethical approval

This study was approved by the Ethics Committee of Chinese Center for Disease Control and Prevention (No: ICDC-2015001). All animals were treated according to the ARRIVE guidelines^44^, the Guidelines of Regulations for the Administration of Laboratory Animals (Decree No. 2 of the State Science and Technology Commission of the People’s Republic of China, 1988) and the Guidelines for Treating Animals Kindly from Ministry of Science and Technology of the People’s Republic of China. All efforts were made to minimize discomfort to the animals.

### Consent to publish

All the authors consent to publish the article in its present form.

## Data Availability

The data supporting the conclusions of this article are included within the article.
